# Shoulder dislocation combined with proximal humerus fracture in children: A case report and literature review

**DOI:** 10.1097/MD.0000000000008977

**Published:** 2017-12-01

**Authors:** Sheng Jin, Haiqing Cai, Yunlan Xu

**Affiliations:** Department of Pediatric Orthopedics, Shanghai Children's Medical Center, Shanghai Jiaotong University School of Medicine, Shanghai, P.R. China.

**Keywords:** child, open reduction, proximal humerus fracture, shoulder dislocation, surgery

## Abstract

**Rationale:**

Proximal humerus fracture occuring simultaneously with dislocation of a shoulder in children is extremely rare, with only a few recent reports of on such cases having been reported.

**Patient concerns:**

A 6-year-old girl fell from a ladder and landed on her dominant right arm with pain in the right shoulder and unable to perform movements; her shoulder did not allow for passive movements as well.

**Diagnoses:**

Proximal humerus fracture combined with shoulder dislocation.

**Interventions:**

The patient was treated with open reduction, elastic stable intramedullary nail (ESIN) fixation, immobilization with U-shape cast and shoulder spica brace.

**Outcomes:**

The patient was pain-free, with full range movement of the injured shoulder and no sign of avascular necrosis in a 2-year follow-up period.

**Lessons:**

We recommend open reduction with ESIN fixation for severely displaced proximal humeral metaphyseal fractures with shoulder dislocation in children. Preoperative bilateral anteroposterior shoulders x-ray is needed to confirm the shoulder location.

## 1 Introduction

Proximal humerus fracture is rarer than other fractures in children, which accounts for 2% to 5.4% of total pediatric fractures.^[1]^ In younger children, most proximal humerus fractures involve the physis with the classification of Salter–Harris type I and II injuries,^[2]^ whereas in the 5 to 12 years old group, metaphyseal fractures are observed more often.^[1]^ The combination of fracture and dislocation of shoulder is extremely rare, except for obstetrical injury.^[3,4]^ A severely displaced fracture, that is, Neer's grade III or IV,^[5]^ is challenging for most surgeons. In existing literatures, there are a few reports of glenohumeral dislocation combined with proximal humerus fracture in pediatric patients. In this study, we present the case of a 6-year-old patient with a proximal humerus fracture (Neer's grade IV) and glenohumeral dislocation, who was treated with open reduction, ESIN fixation, immobilization with U-shape cast, and shoulder spica brace. In a 2-year follow-up period, the patient was pain-free, with full range movement of the right shoulder and no sign of avascular necrosis. The case proves that the ability to accurately and promptly diagnose both the proximal humerus fracture and glenohumeral dislocation is important for selecting appropriate therapeutics and immobilization.

## 2 Case report

**Figure 1:**
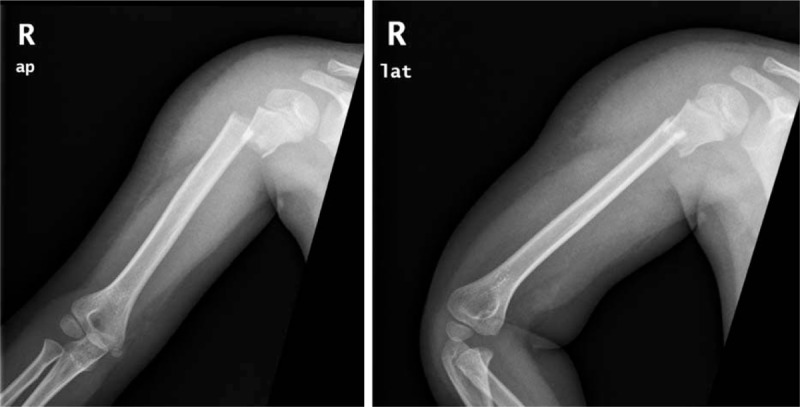
Preoperative anteroposterior and lateral x-ray showing displaced metaphyseal fracture of the right proximal humerus without the sign of shoulder dislocation.

**Figure 2:**
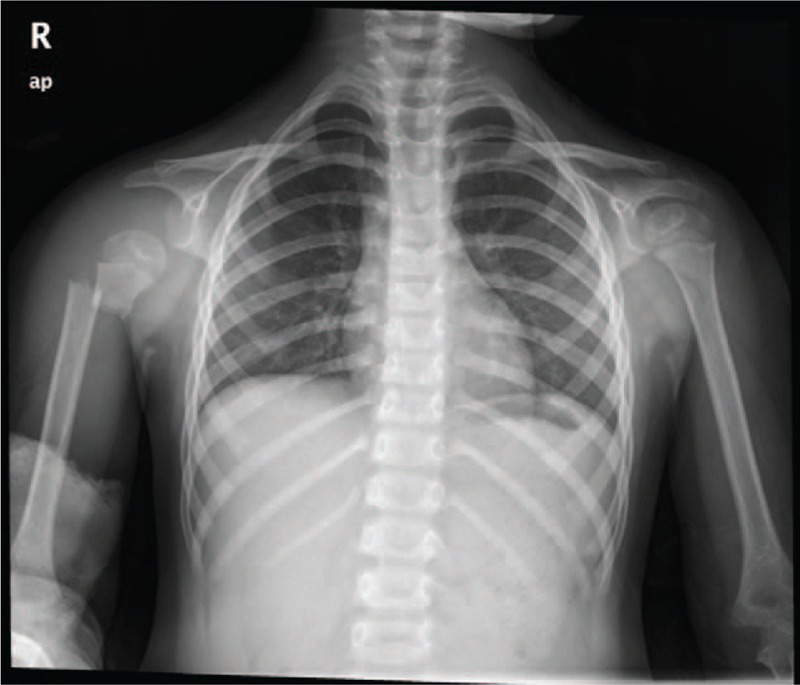
Preoperative bilateral anteroposterior shoulders x-ray showing a combination of right proximal humerus fracture with shoulder dislocation.

A 6-year-old girl was sent to our emergency room after falling from a ladder and landing on her dominant right arm. The patient complained about pain in the right shoulder and was unable to perform movements; her shoulder did not allow for passive movements as well. Physical examination revealed a swollen and painful right shoulder. The right arm was held in slight abduction and external rotation. On palpation, fullness was felt at the anterior aspect of the right shoulder. Both neurological and vascular examinations were normal. Anteroposterior and lateral x-ray of the right humerus and shoulder showed completely displaced proximal humeral metaphyseal fracture (Fig. 1). The anteroposterior view of bilateral shoulders revealed right shoulder dislocation (Fig. 2).

**Figure 3:**
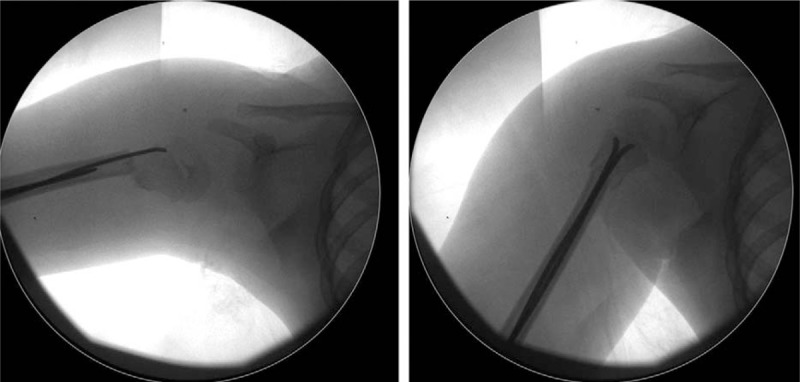
During the operation, the closed reduction failed because of the ESIN could not go through the fracture line into the proximal medullary cavum. After open reduction, the fracture and the humeral head were finally reduced. ESIN = elastic stable intramedullary nail.

**Figure 4:**
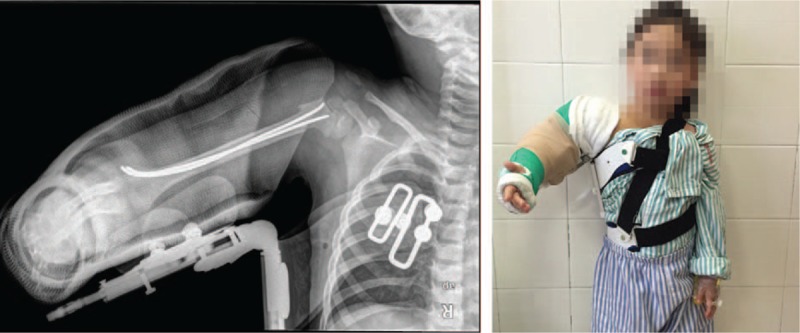
The right upper limb was immobilized with both long arm flexion cast and abduction brace after the wound closure.

**Figure 5:**
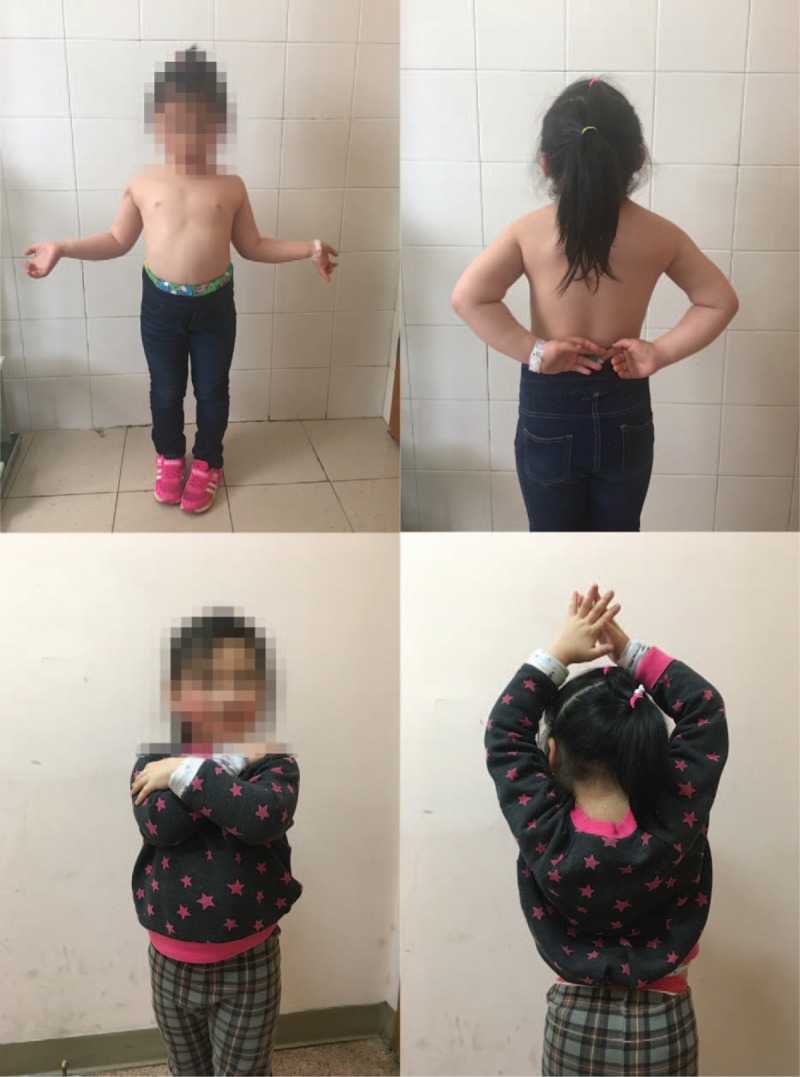
Two years after injury the patient was pain-free and regained full range of motion of the right shoulder.

**Figure 6:**
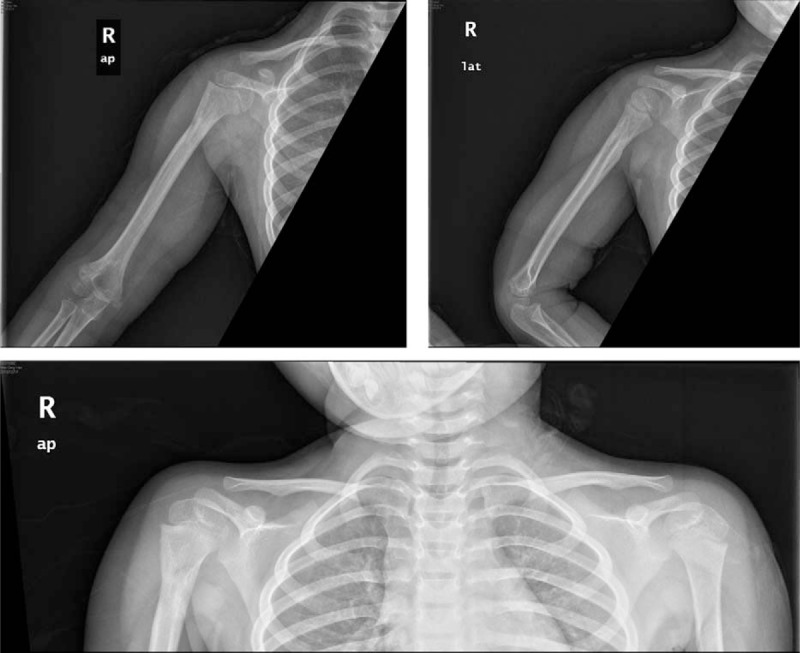
Anteroposterior and lateral x-ray and bilateral anteroposterior shoulders x-ray revealing complete fracture remodeling and no sign of avascular necrosis or any subsequent shoulder dislocation.

A closed reduction and ESIN retrograde fixation of the displaced fracture under anesthesia was attempted, but it was difficult to reduce the fracture by manipulation because of the downward displacement of the proximal fragment along with traction on the distal fragment. The humeral head could not be pushed into the glenoid. Therefore, we performed an open reduction through a deltopectoral approach and the fracture line was exposed. Two nails were introduced into medullary cavum. Then the glenohumeral reduction was performed by manipulation followed by the reduction and internal fixation of the humeral fracture (Fig. 3). After the wound closure, the right upper limb was immobilized with U-shape cast and shoulder spica brace (Fig. 4). Four weeks later, the cast and brace were removed and the patient was commenced on physical therapy in order to increase the range of motion of the shoulder joint. Eight weeks after surgery, x-rays showed abundant bone callus and stable reduction of the right shoulder. In the 2 follow-through years, the patient was pain-free, with full range movement of the right shoulder, normal mobility, and symmetric limb length (Fig. 5). X-rays revealed complete remodeling of the fracture and symmetric bilateral shoulders, with no sign of avascular necrosis (Fig. 6).

## 3 Discussion

Shoulder dislocation is rarely observed in pediatric population. Of all traumatic glenohumeral dislocation patients, <2% of them are <10 years old, and about 20% are between 10 and 20 years old.^[2]^ In an article published in 1956, Rowe reported that only 8 of 500 patients with glenohumeral joint dislocation were <10 years old.^[3]^ Proximal humerus fractures are less rare but are far from common. A handful number of cases of fracture combined with shoulder dislocation in children have been reported in existing literatures. Since Nicastro and Adair first reported in 1982 a case of fracture–shoulder dislocation in a 32-month-old child,^[6]^ only 17 such cases have been documented (Table 1).

**Table 1:**
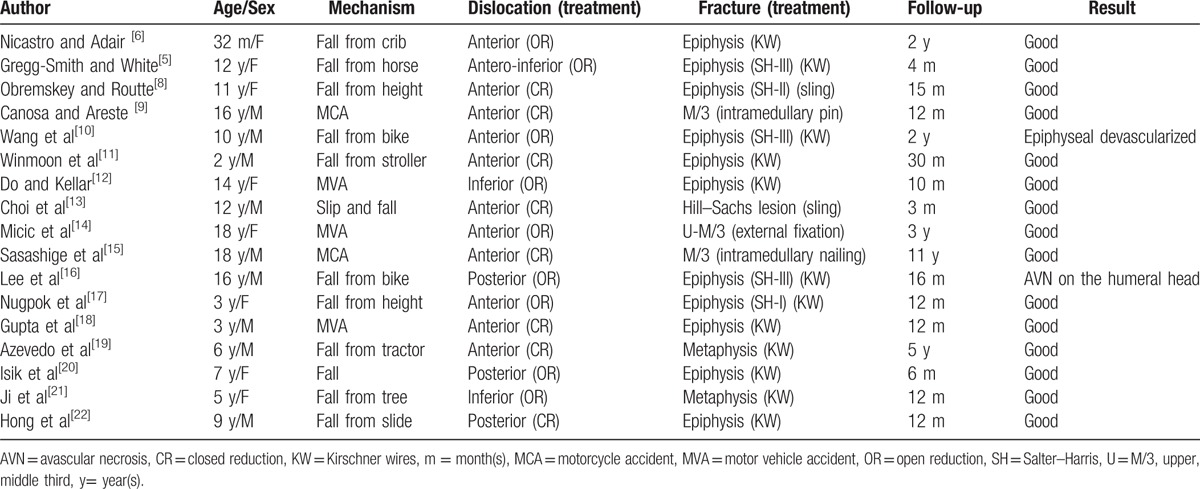
Reported cases of a combination of humerus fracture with ipsilateral shoulder dislocation in children.

Because the proximal humeral epiphyseal plate accounts for about 80% of longitudinal humeral growth,^[1]^ the remodeling of these fractures in children is tremendous. Precise anatomical reduction is not required for functional recovery and most cases can be treated with a sling or hanging arm cast.^[7]^ Nonoperative treatment is appropriate for proximal humeral fractures in children. However, for shoulder dislocation combined with proximal humerus fracture, most authors recommend to do the articular reduction and internal fixation to avoid the complications such as limited range of motion, asymmetry of shoulders, and recurrent dislocations.^[5,6,9–12,15–22]^ Therefore, the treatment of fracture–shoulder dislocation should reach higher requirements.

Open reduction has been reported finally performed on patients of unsuccessful closed reduction because of the soft tissue entrapment like interposed periosteum, deltoid, capsule, or the long head of the biceps tendon.^[16,17,19–21]^ Similarly in our case, we first performed close reduction and ESIN retrograde fixation but failed because the nail was impossible to pass through the displaced fracture line to maintain the reduced position. The glenohumeral joint capsular attachment provides a strong connection to the proximal posteromedial segment of the metaphyseal humeral when the shoulder joint is downward dislocation, joint capsular is broken, and the proximal fragment will drop down followed traction on the distal fragment during the manipulation. So in this case, it was difficult to reduce the fracture by manipulation because of the downward displacement of the proximal fragment. Subsequently, we performed open reduction through the deltopectoral approach. In the open reduction, the displaced fracture can be reduced and the rotator cuff muscles attached to the proximal fragment largely cancel the rotational forces so that the head of humeral can be reduced easily and stably.^[7]^ Therefore, we think that open reduction is needed for irreducible cases such as shoulder dislocation combined with severely displaced humerus fracture.

In our case, we first applied U-shape cast during the operation, but, the C-arm fluoroscopy showed that the shoulder did not reach stable reduction, so the shoulder spica brace was applied to maintain the proper position of the shoulder (Fig. 4). The pectoralis major muscle inserts onto the anteromedial humeral metaphysis. The humeral shaft is pulled anteriorly and the pectoralis major muscle is adducted when a proximal humeral fracture occurs. After the fracture is reduced, the upper limb on abducent position is conducive to reduce the tension of the pectoralis major muscles so that the fracture and the glenohumeral are stabilized. Therefore, we think that it is an effective method to use the shoulder spica brace to keep the glenohumeral joint stable. Again in this case, the cast and the brace were removed at the same time 4 weeks postoperative, the glenohumeral dislocation did not recur. In the following 2 years, the patient had a full range motion of shoulder joint. Neither avascular necrosis of the humeral head nor glenohumeral dislocation was observed.

Moreover, because of proximal humerus fracture combined with shoulder dislocation is a rare injury, some surgeons might just focus on the fracture and neglect the shoulder dislocation. The accurate diagnosis before surgery rely heavily on both palpation and plain radiographs. On palpation, fullness can be felt at the anterior or posterior aspect of the shoulder. On plain radiographs, sometimes anteroposterior and lateral x-ray of humeral just show a displaced fracture and cannot reveal the shoulder dislocation. In our case, we added an anteroposterior x-ray to compare the position of bilateral shoulders, and the right shoulder dislocation was revealed (Fig. 2). Generally speaking, we recommend taking an anteroposterior x-ray of bilateral shoulders routinely for patients with severe proximal humerus fracture to avoid missing diagnosis of the shoulder dislocation.

## 4 Conclusion

Shoulder dislocation combined with proximal humerus fracture in children is rare. Recognition of this type of injury is important. In addition to palpation and anteroposterior and lateral humeral x-ray, we suggest adding bilateral anteroposterior shoulders x-ray routinely to confirm the shoulder location. Open reduction is needed for irreducible dislocation in some severely displaced fractures. Judging from the results of our case, we recommend open reduction with ESIN fixation for the irreducible displaced proximal humeral metaphyseal fractures with shoulder dislocation in children.
